# Endophytic xylariaceous fungi from rice in northern Thailand: discovery of novel species and new host records

**DOI:** 10.3897/mycokeys.120.152187

**Published:** 2025-07-29

**Authors:** Sahar Absalan, Alireza Armand, Ruvishika S. Jayawardena, Nakarin Suwannarach, Jutamart Monkai, Nootjarin Jungkhun, Saisamorn Lumyong, Kevin D. Hyde

**Affiliations:** 1 Department of Biology, Faculty of Science, Chiang Mai University, Chiang Mai 50200, Thailand; 2 Center of Excellence in Microbial Diversity and Sustainable Utilization, Faculty of Science, Chiang Mai University, Chiang Mai 50200, Thailand; 3 Center of Excellence in Fungal Research, Mae Fah Luang University, Chiang Rai 57100, Thailand; 4 School of Science, Mae Fah Luang University, Chiang Rai 57100, Thailand; 5 Office of Research Administration, Chiang Mai University, Chiang Mai 50200, Thailand; 6 Department of Plant Pathology, Faculty of Agriculture at Kamphaeng Saen, Kasetsart University Kamphaeng Saen Campus, Nakhon Pathom 73140, Thailand; 7 Rice Department, Chiang Rai Rice Research Center, Phan, Chiang Rai 57120; 8 Academy of Science, The Royal Society of Thailand, 10300 Bangkok, Thailand; 9 Department of Plant Pathology, College of Agriculture, Guizhou University, Guiyang, Guizhou 550025, China

**Keywords:** fungal endophyte, new taxa, *Oryzasativa*, phylogeny, taxonomy, Xylariomycetidae

## Abstract

Rice (*Oryzasativa* L.) is a major economic crop and a staple food in Asian countries, especially Thailand. Various fungi, including endophytes, are associated with rice and play a significant role in its growth and health. Endophytic xylarialean species are known for their diverse potential roles; however, limited information is available about this group of fungi in relation to rice. Two new species (*Microdochiumoryzicola* and *Nemaniaoryzae*) and three new host records (*Apiosporaintestini*, *A.mukdahanensis*, and *Nemaniaprimolutea*) on rice are introduced in this study. Species identification was based on morphological characteristics and phylogenetic analyses of the combined internal transcribed spacers (ITS), 28S ribosomal RNA (LSU), RNA polymerase II second largest subunit (*rpb*2), β-tubulin (*tub*2), and translation elongation factor 1-alpha (*tef1*-α) loci. Descriptions, illustrations, and phylogenetic analysis results of the new species and new records are provided.

## ﻿Introduction

The term “xylariaceous taxa” refers to the members of subclass Xylariomycetidae, as introduced by Eriksson and Winka (1997). There are three orders within this subclass, including Amphisphaeriales, Delonicicolales, and Xylariales, comprising 40 accepted families (Samarakoon et al. 2022; Hyde et al. 2024). Most xylariaceous species occur in wood, while others can be found on dung and some arthropods in terrestrial and aquatic habitats, as well as acting as plant pathogens (Whalley 1996; Senanayake et al. 2015; Daranagama et al. 2016, 2018; Hyde et al. 2020). The sexual stage of xylariaceous species is well known by their conspicuous stromata, mainly black and thick-walled ascomata with pigmented and aseptate ascospores, and the asexual morph can be observed in both coelomycetes and hyphomycetes (Daranagama et al. 2015, 2016; Maharachchikumbura et al. 2016; Samarakoon et al. 2022). Xylariomycetidae contains species with global distribution and diverse nutritional modes such as endophytes, saprobes, and pathogens (Zhang et al. 2006; Senanayake et al. 2015; U’Ren et al. 2016; Rashmi et al. 2019; Samarakoon et al. 2023).

Over the past half-century, research has unveiled a remarkable diversity of xylariaceous endophytes (Petrini and Petrini 1985). Endophytic fungi live inside plant tissues without causing symptoms (Arnold 2007). They perform various functions in host plants, including enhancing resistance to pathogens, regulating host responses to abiotic stressors, and generating secondary metabolites (Rogers 2000; Arnold et al. 2003; Mejía et al. 2008; Hartley et al. 2015). Xylariaceous endophytes have been discovered in a wide range of plant groups, including flowering plants (monocotyledons and dicotyledons), conifers, and mosses across different climate zones, with a particular prevalence in tropical regions (Brunner and Petrini 1992; Okane et al. 2012; Davey et al. 2014; Ikeda et al. 2014). Thailand is regarded as one of the regions with a high richness of xylarialean taxa (Rogers 2000; Samarakoon et al. 2023). For instance, xylariaceous endophytes, including *Nemania* and *Xylaria* as major genera, have been isolated from many tropical plants in Thailand (Okane et al. 2012). Additionally, *Daldiniaeschscholtzii* has been reported as the most frequent xylariaceous endophyte in Thai plants (Chareprasert et al. 2010, 2012), such as leaves of *Tectonagrandis* (teak) (Mekkamol 1998). Some studies have shown a high diversity of endophytic species of Xylariaceae in bamboo (*Bambusavulgaris*), wild banana (*Musaacuminata*), wild ginger (*Amomumsiamense*), *Garcinia* spp., and *Dendrobium* spp. in Thailand (Bussaban et al. 2001; Photita et al. 2001; Phongpaichit et al. 2006; Ma et al. 2022). Although several studies on xylariaceous endophytes have been conducted in Thailand, there is still limited knowledge of their association with rice plants. Thus, this study aimed to fill this gap. It can provide valuable insights for future studies due to the significance of Thailand’s primary crop and the beneficial role of endophytic xylarialean species.

## ﻿Materials and methods

### ﻿Sample collection, isolation, and examination

Healthy tissue parts (leaves and panicles) of glutinous and jasmine rice cultivars were collected from Chiang Rai Province, Thailand, from November to December 2021. Samples enclosed in plastic bags were transported to the laboratory with labels containing collection details. A surface sterilization method (Johnson and Whitney 1989), with some modifications, was carried out to isolate endophytic fungi. Each plant part was washed thoroughly under running tap water for several minutes and then cut into 1 × 1 cm^2^ pieces, which were soaked in 70% ethanol for 1 min, followed by 15 min in 10% sodium hypochlorite solution, rinsed three times with sterile distilled water, and transferred to sterile filter paper. The segments were then placed onto 9 cm petri dishes containing potato dextrose agar (PDA) medium fortified with tetracycline to prevent bacterial growth. The plates were incubated at 26 °C for five days in the dark. After incubation, the mycelia growing from the tissue segments were individually subcultured onto fresh PDA medium (Senanayake et al. 2020). The hyphal tip technique was used as described by Tutte (1969) to obtain pure cultures of each isolate. Dry fungal cultures were prepared using one-month-old colonies grown on PDA. A solution was made by dissolving 2 g of agar powder in 200 ml of distilled water and mixing with 10 ml of glycerol. The mixture was heated until the agar fully dissolved and then allowed to cool for 5–10 min. The cooled solution was carefully poured over the fungal culture, which had been transferred from its original petri dish to a sterile surface. The coated culture was then left to air dry at room temperature. Microscopic and macroscopic features were examined for morphological identification using a stereomicroscope and compound microscope (Nikon Eclipse 80i) equipped with a digital camera. Measurements were obtained using the Tarosoft® Image Frame Work program, and further modifications were made using Adobe Photoshop version 21.1.3 (Adobe, USA). Dried and living cultures were deposited in the Mae Fah Luang University Fungarium (MFLU) and Mae Fah Luang University Culture Collection (MFLUCC), respectively. Index Fungorum (https://www.indexfungorum.org/names/names.asp) and Faces of Fungi (Jayasiri et al. 2015) numbers were obtained for novel taxa.

### ﻿DNA extraction, PCR amplification, and sequencing

Two hundred milligrams of two-week-old mycelia were used to extract genomic DNA following the protocols of the OMEGA E.Z.N.A.® Forensic DNA Kit. Five loci, including internal transcribed spacers (ITS), 28S large subunit ribosomal RNA (LSU), RNA polymerase II second largest subunit (*rpb*2), β-tubulin (*tub*2), and translation elongation factor 1-alpha (*tef1*-α), were amplified by polymerase chain reaction (PCR) using appropriate primers. The PCR thermal cycle program and primers used in this study are listed in Table 1. The PCR mixture consisted of 12.5 μl of 2× Power Taq PCR MasterMix (a ready-to-use solution containing Taq DNA polymerase, dNTPs, and optimized buffer), 9.5 μl of deionized water, 1 μl each of forward and reverse primers (10 pM), and 1 μl of genomic DNA. After amplification, the presence of positive amplicons was confirmed using agarose gel electrophoresis. The gels were stained with Cybergreen and visualized under UV light using a molecular imaging system. Positive PCR products were sent to SolGent Co., Republic of Korea, for purification and sequencing using the same primer pairs.

**Table 1.** Primers and PCR conditions used in this study.

Locus	Primers	PCR conditions	References
ITS	ITS5/ITS4	94 °C 3 min; 35 cycles of 94 °C 45 s, 56 °C 1 min, 72 °C 1 min; 72 °C 10 min	White et al. 1990
LSU	LROR/LR5	94 °C 3 min; 35 cycles of 94 °C 30 s, 55 °C 50 s, 72 °C 90 s; 72 °C 10 min	Vilgalys and Hester 1990
*tef1*-α	EF-1/EF-2	94 °C 90 s; 35 cycles of 94 °C 45 s, 55 °C 45 s, 72 °C 1 min; 72 °C 10 min	Carbone and Kohn 1999
*tub*2	T1/Bt2b	94 °C 3 min; 35 cycles of 94 °C 30 s, 56 °C 30 s, 72 °C 1 min; 72 °C 10 min	Glass and Donaldson 1995
*rpb*2	RPB2–5F2/RPB2–7cR	94 °C 90 s; 40 cycles of 94 °C 30 s, 55 °C 30 s, 72 °C 2 min; 72 °C 10 min	Liu et al. 1999

### ﻿Phylogenetic analyses

Phylogenetic analyses were conducted following the methods outlined in Dissanayake et al. (2020). Sequence data for all loci were subjected to a BLASTn search to retrieve related sequences from the NCBI (National Center for Biotechnology Information, https://blast.ncbi.nlm.nih.gov/Blast.cgi) database. Individual loci were aligned using MAFFT version 7.036 (http://mafft.cbrc.jp/alignment/server/large.html; Katoh et al. 2019) with default parameters. Prior to phylogenetic analyses, the sequence alignments were manually adjusted as necessary using BioEdit version 7.0.5.2 (Hall 1999).

The combined alignment underwent Maximum Likelihood (ML) analysis using RAxML-HPC2 on XSEDE (version 8.2.8) (Stamatakis et al. 2008; Stamatakis 2014) via the CIPRES Science Gateway platform (Miller et al. 2010). The analysis employed the GTRGAMMA model of evolution, and bootstrap supports were generated based on 1,000 replicates. Bayesian posterior probabilities (PP) were calculated using MrBayes version 3.1.2 (Huelsenbeck and Ronquist 2001) through Markov Chain Monte Carlo (MCMC) sampling, employing four simultaneous Markov chains, running for 1,000,000 generations, and sampling every 100^th^ generation. The initial 25% of trees from the burn-in phase were discarded, and the remaining 75% were used to calculate posterior probabilities. Phylogenetic trees were visualized using FigTree version 1.4.0 (Rambaut 2012) and edited using Adobe Illustrator CC 22.0.0 (Adobe Systems, USA).

## ﻿Results

From 24 samples collected across five districts, eight fungal isolates were recovered, all classified within the Xylariomycetidae. Micro-morphological characteristics and phylogenetic analyses led to the discovery of two new species (*Microdochiumoryzicola* and *Nemaniaoryzae*) and the identification of three new host records (Table 2).

**Table 2.** Details of the species obtained in this study.

Species	Strain number	Plant tissue part	Location	Cultivar
*Apiosporaintestini*	MFLUCC 24-0510	leaf	Mueang Phan Sub-district, Phan District, Chiang Rai Province	RD15
*Apiosporamukdahanensis*	MFLUCC 24-0511	panicle	Huai Sak Sub-district, Mueang Chiang Rai District, Chiang Rai Province	RD15
*Daldiniaeschscholtzii*	MFLUCC 24-0504	panicle	Mueang Phan Sub-district, Phan District, Chiang Rai Province	RD6
	MFLUCC 24-0505	panicle	Thung Ko sub-district, Wiang Chiang Rung District, Chiang Rai Province	Nan59
	MFLUCC 24-0506	panicle	San Sai Ngam Sub-district, Thoeng District, Chiang Rai Province	SPT1
*Microdochiumoryzicola*	MFLUCC 24-0509	leaf	Doi Luang District, Chiang Rai Province	CP 888
*Nemaniaprimolutea*	MFLUCC 24-0507	panicle	San Sai Ngam Sub-district, Thoeng District, Chiang Rai Province	SPT1
*Nemaniaoryzae*	MFLUCC 24-0508	panicle	Mueang Phan Sub-district, Phan District, Chiang Rai Province	RD6

### ﻿Phylogenetic analysis

The phylogram of *Daldinia*, including 46 strains, was generated from ML analysis based on concatenated four-locus (ITS, LSU, *rpb*2, *tub*2) sequence data, which comprised 3,942 characters after alignment. The best-scoring RAxML tree, with a final ML optimization likelihood value of –18009.449198, is presented. The matrix contained 1,258 distinct alignment patterns, with 46% of undetermined characters or gaps. Estimated base frequencies were as follows: A = 0.238507, C = 0.256708, G = 0.258390, T = 0.246395; substitution rates were AC = 1.331536, AG = 4.448940, AT = 1.375914, CG = 1.061357, CT = 7.325168, GT = 1.000000. The gamma distribution shape parameter alpha was 0.195267. The Bayesian tree converged at the 1,000,000^th^ generation with an average standard deviation of split frequencies of 0.052336.

The phylogram of *Nemania*, including 43 strains, was generated from ML analysis based on concatenated four-locus (ITS, LSU, *rpb*2, *tub*2) sequence data, which comprised 2,456 characters after alignment. The best-scoring RAxML tree, with a final ML optimization likelihood value of –19593.464797, is presented. The matrix contained 984 distinct alignment patterns, with 24.01% of undetermined characters or gaps. Estimated base frequencies were as follows: A = 0.252794, C = 0.252919, G = 0.262664, T = 0.231622; substitution rates were AC = 1.337793, AG = 5.997557, AT = 1.050824, CG = 1.258166, CT = 8.646548, and GT = 1.000000. The gamma distribution shape parameter alpha was 0.199517. The Bayesian tree converged at the 1,000,000^th^ generation with an average standard deviation of split frequencies of 0.025689.

The phylogram of Microdochiaceae, including 72 strains, was generated from ML analysis based on concatenated three-locus (ITS, LSU, *rpb*2) sequence data, which comprised 2,048 characters after alignment. The best-scoring RAxML tree, with a final ML optimization likelihood value of –15255.408941, is presented. The matrix contained 689 distinct alignment patterns, with 14.02% of undetermined characters or gaps. Estimated base frequencies were as follows: A = 0.262262, C = 0.228560, G = 0.263931, and T = 0.245247; substitution rates were AC = 1.072625, AG = 4.845426, AT = 1.419528, CG = 0.964509, CT = 7.796411, and GT = 1.000000. The gamma distribution shape parameter alpha was 0.149445. The Bayesian tree converged at the 1,000,000^th^ generation with an average standard deviation of split frequencies of 0.017893.

The phylogram of *Apiospora*, including 153 strains, was generated from ML analysis based on concatenated three-locus (ITS, *tef1*-α, *tub*2) sequence data, which comprised 1,343 characters after alignment. The best-scoring RAxML tree, with a final ML optimization likelihood value of –18735.939045, is presented. The matrix contained 828 distinct alignment patterns, with 19.41% of undetermined characters or gaps. Estimated base frequencies were as follows: A = 0.226457, C = 0.276708, G = 0.229953, and T = 0.266881; substitution rates were AC = 1.277370, AG = 3.702079, AT = 1.247535, CG = 1.072981, CT = 4.623924, and GT = 1.000000. The gamma distribution shape parameter alpha was 0.342877. The Bayesian tree converged at the 1,000,000^th^ generation with an average standard deviation of split frequencies of 0.026022.

### ﻿Taxonomy

#### ﻿*Apiospora* Sacc., Atti Soc. Veneto-Trent. Sci. Nat., Padova, Sér. 4 4: 85 (1875)

*Apiospora*, with the type species *A.montagnei*, was introduced by Saccardo (1875). Apiosporaceae was later established by Hyde et al. (1998) to accommodate arthrinium-like taxa, which currently comprises *Apiospora*, *Arthrinium*, *Neoarthrinium*, and *Nigrospora* (Hyde et al. 2020; Konta et al. 2021; Jiang et al. 2022; Samarakoon et al. 2022). The phylogenetic relationship between *Apiospora* and *Arthrinium* has been a topic of frequent debate (Crous and Groenewald 2013; Réblová et al. 2016; Wang et al. 2018; Jiang et al. 2019), which subsequently led to the synonymization of most *Arthrinium* species under *Apiospora* (Pintos and Alvarado 2021). *Apiospora* species are distributed globally in terrestrial and aquatic habitats (Ramos et al. 2010; Hyde et al. 2020; Kwon et al. 2022). The majority of species are recognized as saprobes and endophytes in a various range of host plants, predominantly found in Poaceae. Additionally, some species are significant plant pathogens that cause substantial harm to economically important plants (Mavragani et al. 2007; Dai et al. 2016; Aiello et al. 2018; Wang et al. 2018; Yin et al. 2020; Pintos and Alvarado 2021).

##### ﻿ Apiospora intestini

Taxon classificationFungiXylarialesApiosporaceae

(Kajale, Sonawane and Roh. Sharma) Pintos and P. Alvarado, Fungal Syst. Evol. 7: 206 (2021)

323E4603-959A-5933-BC06-D45C1A01AF4D

Index Fungorum: IF837744

Facesoffungi Number: FoF17637

Fig. 1

###### Description.

*Endophytic* from healthy leaf of *Oryzasativa*. **Sexual morph**: not observed. **Asexual morph**: ***Hyphae*** 4–5.5 μm wide, septate, thick-walled, hyaline to brown. ***Conidiophores***, ***conidiogenous cells***, and ***conidia*** not observed.

**Figure 1.**
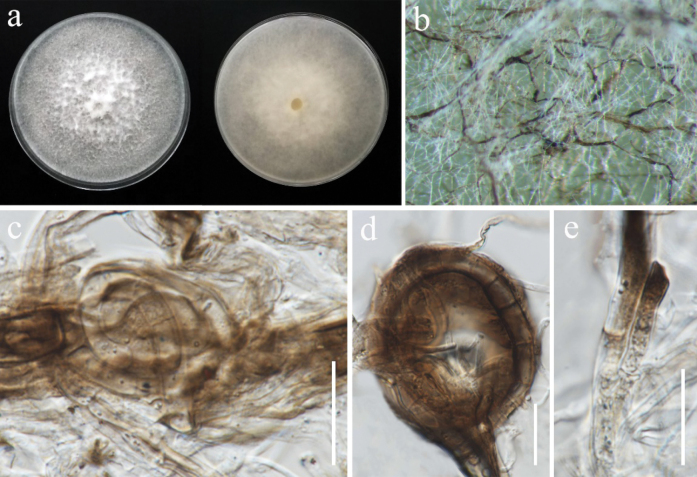
*Apiosporaintestini* (MFLUCC 24-0510, new host record). **a.** Front and reverse of the colony on PDA; **b–e.** Hyphae. Scale bars: 20 μm (**c–e**).

###### Culture characteristics.

Colonies on PDA reaching 86–90 mm in diameter after 7 days at 28 °C, white, medium dense with some immersed dark brown mycelia, cottony; reverse white.

###### Material examined.

Thailand • Chiang Rai Province, Phan District, Mueang Phan Subdistrict, from healthy tissue part of rice leaf, 5 January 2022, Sahar Absalan (NS50-1a = MFLU 25-0029) (living culture MFLUCC 24-0510).

###### GenBank numbers.

MFLUCC 24-0510: ITS = PV235258, *tef1*-α = N/A, *tub*2 = PV275687.

###### Notes.

**Figure 2.**
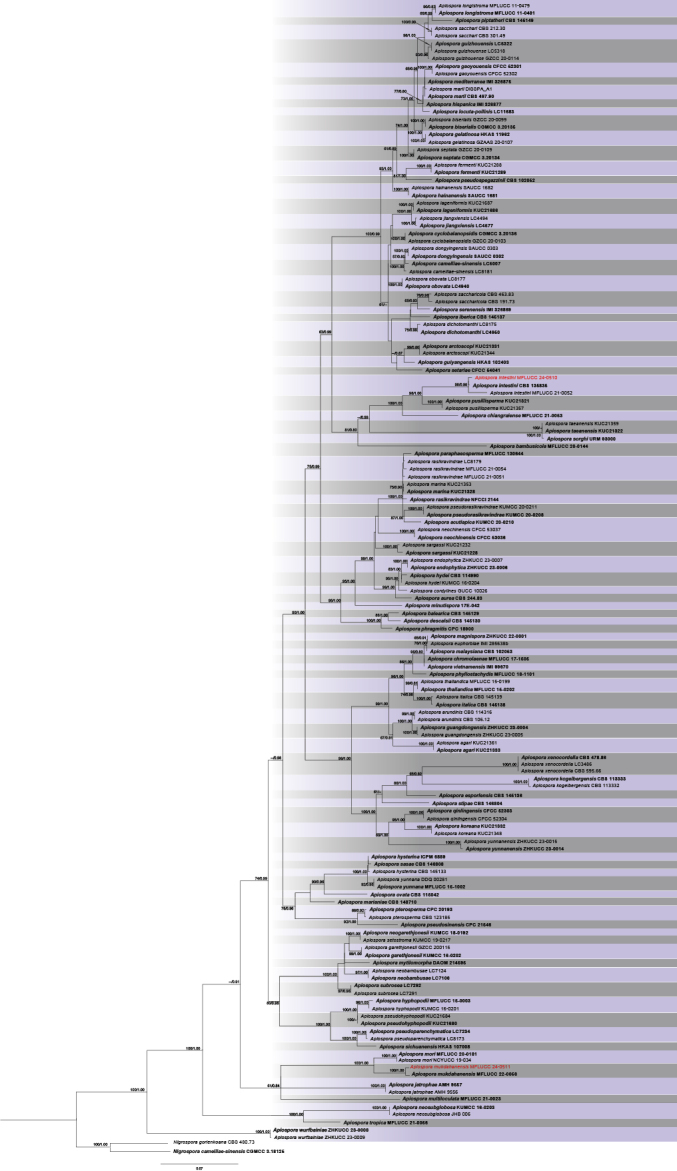
Phylogram of ML analysis based on combined ITS, *tef1*-α, and *tub*2 sequence data. ML bootstrap support values equal to or higher than 60% and Bayesian probability values (PP) equal to or above 0.80 are given at the nodes (ML/PP). The tree is rooted to *Nigrosporagorlenkoana* (CBS 480.73) and *N.camelliae-sinensis* (CGMCC 3.18125). The isolate from the current study is highlighted in red, and type strains are indicated in bold black.

*Apiosporaintestini* was introduced by Crous et al. (2015) as *Arthriniumgutiae* isolated from the gut of a grasshopper. It was also isolated as a saprobe from bamboo in Chiang Rai Province, Thailand (Tian et al. 2021a). Phylogenetic analysis of a concatenated ITS, *tef1*-α, and tub sequence dataset indicated that our strain (MFLUCC 24-0510) has a close affinity with *A.intestini* with 98% ML and 0.98 PP support (Fig. 2). However, we were not able to compare the morphological characteristics because our isolate remained sterile in culture. Therefore, we report this species as a new host record from rice.

##### ﻿ Apiospora mukdahanensis

Taxon classificationFungiXylarialesApiosporaceae

Monkai and Phookamsak, Diversity 14(no. 918): 11 (2022)

51565323-3D95-5AAF-9B6A-9C11A1B83379

Index Fungorum: IF559912

Facesoffungi Number: FoF12853

Fig. 3

###### Description.

*Endophytic* from healthy panicle of *Oryzasativa*. **Sexual morph**: not observed. **Asexual morph**: ***Conidiophores*** 2.5–4 μm wide, basauxic, cylindrical, septate, straight or flexuous, sometimes reduced to conidiogenous cells, hyaline. ***Conidiogenous cells*** 7–21.5 × 4.5–6 μm (*x̄* = 13 × 5 µm, *n* = 10), cylindrical to subcylindrical, lageniform or ampulliform, pale brown to hyaline. ***Conidia*** 5–7 × 4–5.5 μm (*x̄* = 6.5 × 5 µm, *n* = 25), globose to subglobose, lenticular to side view with a pale longitudinal germ slit, thick-walled, brown to dark brown.

**Figure 3.**
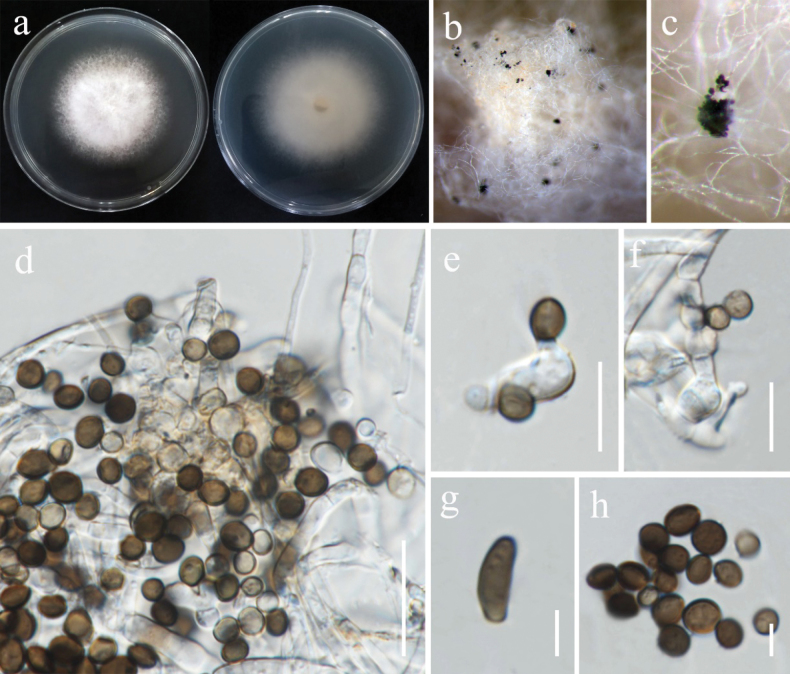
*Apiosporamukdahanensis* (MFLUCC 24-0511, new host record). **a.** Front and reverse of the colony on PDA; **b, c.** Conidiomata formed on PDA; **d.** Conidiophores, conidiogenous cells, and conidia; **e, f.** Conidiogenous cells **g.** Sterile cell; **h.** Conidia. Scale bars: 20 μm (**d**);10 μm (**e–f**); 5 μm (**g–h**).

###### Culture characteristics.

Colonies on PDA reaching 46–48 mm in diameter after 7 days at 28 °C, white, becoming pale orange with age, circular, floccose to cottony; reverse dull white.

###### Material examined.

Thailand • Chiang Rai Province, Mueang Chiang Rai District, Huai Sak Subdistrict, from healthy tissue part of rice panicle, 17 December 2021, Sahar Absalan (HS88-2b = MFLU 25-0030) (living culture MFLUCC 24-0511).

###### GenBank numbers.

MFLUCC 24-0511: ITS = PV235259, *tef1*-α = PV275673, *tub*2 = N/A.

###### Notes.

Based on the morphological and molecular data, the isolate MFLUCC 24-0511 was identified as a representative of *Apiosporamukdahanensis* with 100% ML and 1.00 PP bootstrap support (Fig. 2). *Apiosporamukdahanensis* was described by Monkai et al. (2022) and isolated from dead leaves of bamboo in Mukdahan Province, Thailand. Morphologically, our strain closely resembles the type strain MFLUCC 22-0056. However, our observations revealed the presence of sterile cells, which were not reported in their collection (Monkai et al. 2022). This study describes this species as a new host record from rice.

#### ﻿*Daldinia* Ces. and De Not., Comm. Soc. crittog. Ital. 1(fasc. 4): 197 (1863)

*Daldinia* was introduced by Cesati and De Notaris (1863) with the type species *D.concentrica* (Bolton) Ces. and De Not. Initially, the genus was placed in the Xylariaceae family; however, subsequent research based on a multi-locus phylogeny analysis conducted by Wendt et al. (2018) demonstrated that *Daldinia* belongs to Hypoxylaceae. The monograph of *Daldinia* provided by Stadler et al. (2014) examined more than 1,000 specimens through various taxonomic approaches and chemotaxonomic data. The species in this genus encompass saprobes causing white rot of dead wood and endophytes (Johannesson et al. 2000; Guidot et al. 2003; Stadler et al. 2014).

##### ﻿ Daldinia eschscholtzii

Taxon classificationFungiXylarialesHypoxylaceae

(Ehrenb.) Rehm, Annls. Mycol. 2(2): 175 (1904)

DB0A6704-675F-5583-BF7D-E3BD6844FD1A

Index Fungorum: IF544992

Facesoffungi Number: FoF02990

Fig. 4

###### Description.

*Endophytic* from healthy panicle of *Oryzasativa*. **Sexual morph**: not observed. **Asexual morph**: hyphomycetous. ***Conidiophores*** 1–2.3 × 0.9–1.7 µm (*x̄* = 2 × 1.4 µm, *n* = 10), mononematous, dichotomously or trichotomously branched, with *Nodulisporium*-like branching pattern, bearing 1–3 conidiogenous cells from each whorl, hyaline. ***Conidiogenous cells*** 2.6–4 × 1.5–4 µm (*x̄* = 3.5 × 2 µm, *n* = 10), holoblastic, terminal or intercalary, cylindrical, with rounded apices, hyaline. ***Conidia*** 3–5.8 × 2.6–4.2 µm (*x̄* = 4 × 3.3 µm, *n* = 30), smooth, obovoid to ellipsoid, aseptate, mostly with flat base, hyaline.

**Figure 4.**
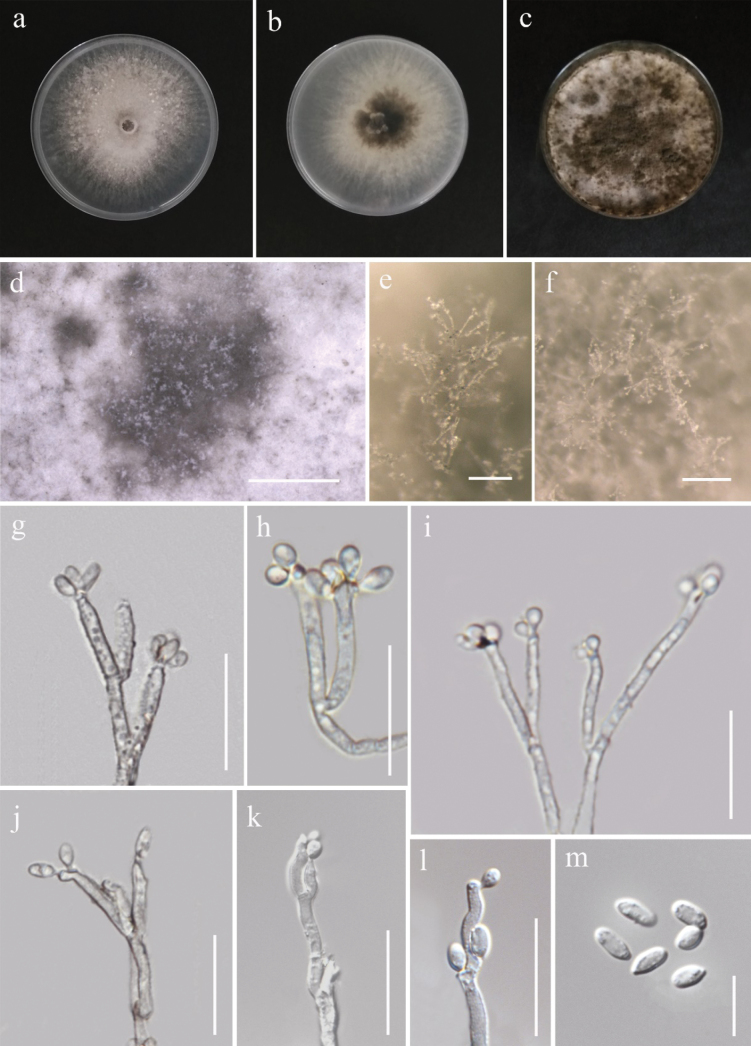
*Daldiniaeschscholtzii* (MFLUCC 24-0504). **a, b.** Front and reverse of the colony on PDA after a week **c.** Colony appearance on PDA after 4 weeks with dark green patches; **d–f.** Sporulation of colony on PDA; **g–j.** Conidial attachments and conidiogenous cells showing *Nodulisporium*-like branching pattern; **k, l.** Conidial attachments and conidiogenous cells showing sporothrix-like branching pattern; **m.** Conidia. Scale bars: 500 µm (**d**); 200 µm (**e–f**); 20 µm (**g–l**); 10 µm (**m**).

###### Culture characteristics.

Colonies on PDA reaching 63–65 mm in diameter after 7 days at 27 °C, initially white with a diffuse margin. Becoming grayish olive green with dull green patches; reverse black at the center and whitish gray at the periphery.

###### Material examined.

Thailand • Chiang Rai Province, Phan District, from healthy tissue of rice panicle, 25 October 2021, Nootjarin Jungkhun (NS11-1a = MFLU 25-0025); (living culture MFLUCC 24-24-0504).

###### GenBank numbers.

MFLUCC 24-0504: ITS = PV235051, LSU = PV235061, *rpb*2 = PV275678, *tub*2 = PV275684; MFLUCC 24-0505: ITS = PV235052, LSU = PV235062, *rpb*2 = PV275679, *tub*2 = N/A; MFLUCC 24-0506: ITS = PV235053, LSU = N/A, *rpb*2 = PV275680, *tub*2 = N/A.

###### Notes.

**Figure 5.**
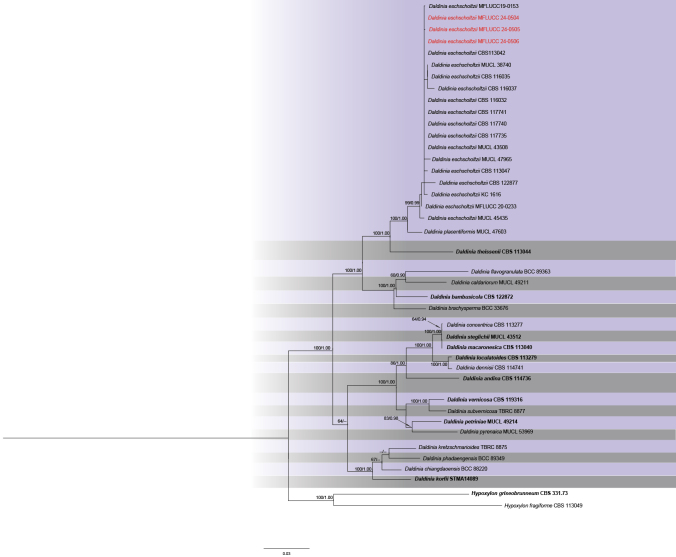
Phylogram of ML analysis based on combined ITS, LSU, *rpb*2, and *tub*2 sequence data. ML bootstrap support values equal to or higher than 60% and Bayesian probability values (PP) equal to or above 0.90 are given at the nodes (ML/PP). The tree is rooted to *Hypoxylonfragiforme* (CBS 113049) and *H.griseobrunneum* (CBS 331.73). The isolates from the current study are highlighted in red, and type strains are indicated in bold black.

Based on the morphological and molecular data, all three strains (MFLUCC 24-0504, MFLUCC 24-0505, and MFLUCC 24-0506) were identified as *Daldiniaeschscholtzii* with 99% ML and 0.99 PP bootstrap support (Fig. 5). Isolates MFLUCC 24-0504, MFLUCC 24-0505, and MFLUCC 24-0506 were obtained from panicles of both glutinous and jasmine rice collected from various locations in Chiang Rai Province of Thailand (Table 2).

#### ﻿*Microdochium* Syd. and P. Syd., Annls mycol. 22(3/6): 267 (1924)

*Microdochium* was introduced by Sydow (1924) with *M.phragmitis* as the type species. Previous research based on morphology indicated that *Microdochium* is classified within the family Amphisphaeriaceae (Von Arx 1984; Jaklitsch and Voglmayr 2012). Later, Hernández-Restrepo et al. (2016) proposed the new family Microdochiaceae to accommodate *Idriella*, *Microdochium*, and *Selenodriella*, as these three genera formed a unique lineage within the Xylariales. Species of *Microdochium* have a diverse host range and are commonly categorized as endophytes, plant pathogens, and saprobes based on their characteristics and ecological roles (Glynn et al. 2005; Jewell and Hsiang 2013; Hiruma et al. 2018; Liang et al. 2019; Huang et al. 2020).

##### ﻿ Microdochium oryzicola

Taxon classificationFungiAmphisphaerialesAmphisphaeriaceae

S. Absalan, S. Lumyong and K.D. Hyde sp. nov.

BB103FEE-83C1-5E4B-8F0D-36BCA40592CB

Index Fungorum: IF903561

Facesoffungi Number: FoF17636

Fig. 6

###### Holotype.

MFLU 25-0028

###### Etymology.

*Oryzicola* refers to the host genus *Oryza* from which it was isolated.

**Figure 6.**
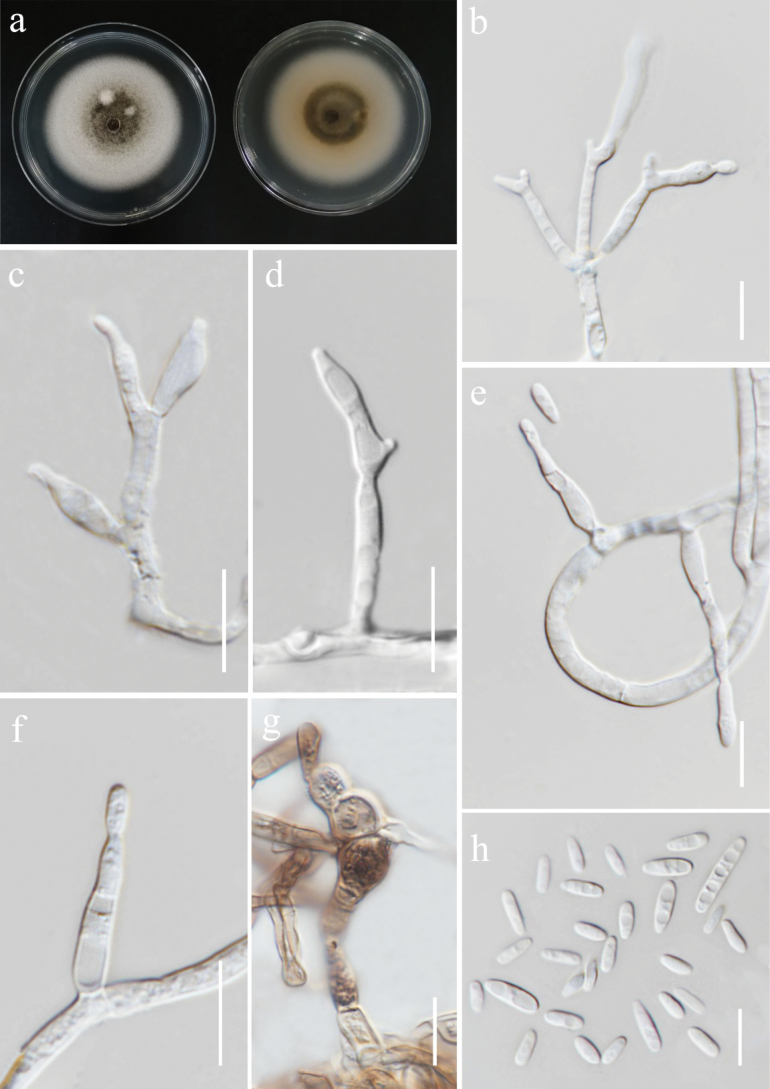
*Microdochiumoryzicola* (MFLUCC 24-0509, ex-type). **a.** Front and reverse of the colony on PDA; **b–f.** Conidiophores and conidiogenous cells; **g.** Chlamydospores; **h.** Conidia. Scale bars: 10 μm (**b–h**).

###### Description.

*Endophytic* from healthy leaf of *Oryzasativa*. **Sexual morph**: not observed. **Asexual morph**: hyphomycetous. ***Mycelium*** 2.5–4.5 μm wide, mostly superficial, branched, septate, smooth, hyaline. ***Conidiophores*** 22–63 × 2–4.5 µm (*x̄* = 37 × 3.5 µm, *n* = 10), usually reduced to conidiogenous cells, mono- or bi-verticillate, branched, smooth-walled, hyaline. ***Conidiogenous cells*** 6–24 × 2–5 µm (*x̄* = 15 × 3 µm, *n* = 20), cylindrical, lageniform to ampulliform, terminal or intercalary, mono- or polyblastic, hyaline. ***Conidia*** 6–16 × 2.5–4 µm (*x̄* = 8.5 × 3.3 µm, *n* = 30), solitary, aseptate, cylindrical to clavate, obovoid, guttulate, hyaline. ***Chlamydospores*** abundant, globose to subglobose, sometimes irregular, in chains, thick-walled, pale brown to brown.

###### Culture characteristics.

Colonies on PDA reaching 65–67 mm in diameter after 7 days at 28 °C, dark olivaceous grey in the center and white to the periphery, circular, fluffy aerial mycelium; reverse buff with olivaceous grey in the center.

###### Material examined.

Thailand • Chiang Rai Province, Doi Luang District, from healthy tissue part of rice leaf, 7 February 2022, Nootjarin Jungkhun, (NS62-1 = MFLU 25-0028); (living culture MFLUCC 24-0509).

###### GenBank numbers.

MFLUCC 24-0509: ITS = PV241406, LSU = PV241407, *rpb*2 = PV275683.

###### Notes.

**Figure 7.**
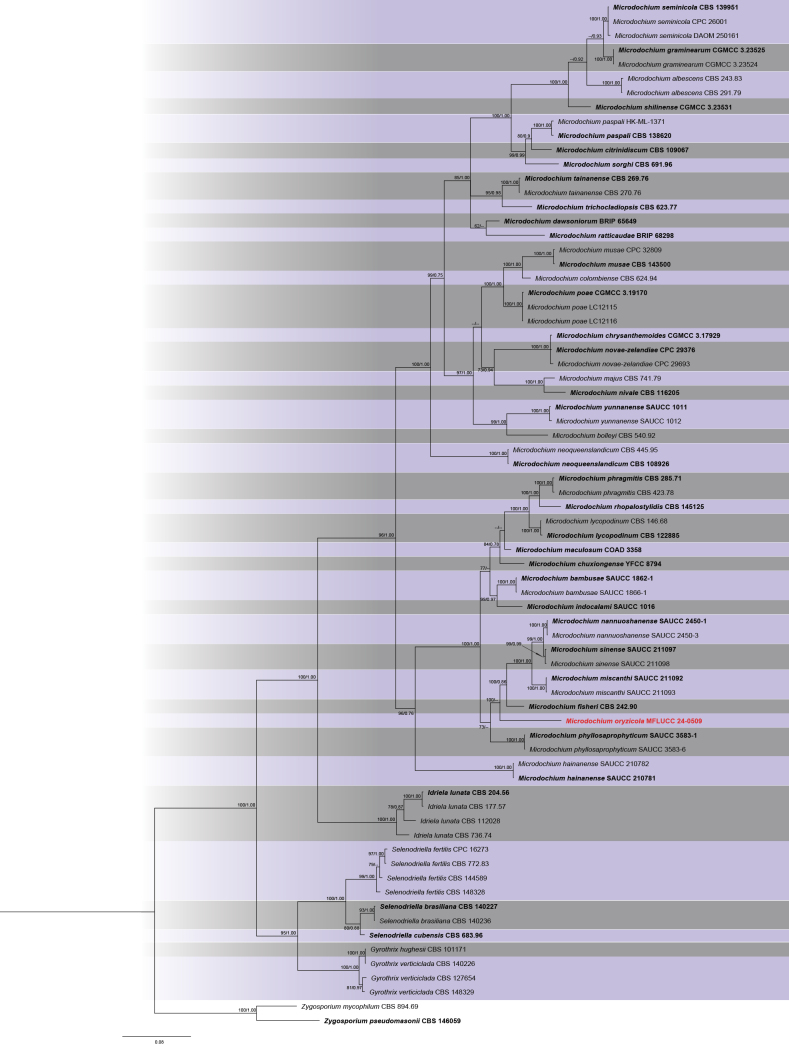
Phylogram of ML analysis based on combined ITS, LSU, and *rpb*2 sequence data. ML bootstrap support values equal to or higher than 60% and Bayesian probability values (PP) equal to or above 0.80 are given at the nodes (ML/PP). The tree is rooted to *Zygosporiumpseudomasonii* (CBS 146059) and *Z.mycophilum* (CBS 894.69). The isolate from the current study is highlighted in red, and type strains are indicated in bold black.

Phylogenetic analysis of combined ITS, LSU, and *rpb*2 sequences revealed that our strain (MFLUCC 24-0509) is from a distinct branch, constituting a well-supported lineage (100% ML) separate from its sister clade, which includes *Microdochiumnannuoshanense*, *M.sinense*, *M.miscanthi*, and *M.fisheri* (Fig. 7). *Microdochiumoryzicola* is morphologically distinct from its closely related species, *M.fisheri*, particularly in the characteristics of the conidiophores and conidiogenous cells. *Microdochiumoryzicola* has branched mono- or biverticillate conidiophores with mainly lageniform to ampulliform conidiogenous cells, whereas *M.fisheri* is characterized by bifurcate conidiophores and cylindrical, sympodial, conidiogenous cells (Hernández-Restrepo et al. 2016). A comparison of the nucleotide differences between *M.oryzicola* and *M.fisheri* revealed 2.88% (across 520 nucleotides, 9 gaps), 1.48% (across 810 nucleotides, 2 gaps), and 12.7% (across 697 nucleotides, without gaps) base pair differences in the ITS, LSU, and *rpb*2 gene regions, respectively. Thus, *M.oryzicola* is introduced as a new species.

#### ﻿*Nemania* Gray, Nat. Arr. Brit. Pl. (London) 1: 516 (1821)

*Nemania* was established by Gray (1821) and belongs to the family Xylariaceae. Previously, some members of *Nemania* were considered synonyms of *Hypoxylon* (Miller 1961). However, Tang et al. (2007) presented phylogenetic evidence from ITS and *rpb*2 sequences that affirmed the differentiation of *Nemania* from *Hypoxylon*. *Nemania* species are mainly regarded as saprophytic organisms and commonly found in decaying wood (Granmo et al. 1999; Ju and Rogers 2002; Wijayawardene et al. 2017; Daranagama et al. 2018). However, there have been documented instances of these fungi existing as endophytes within different plant species (Kumarihamy et al. 2019; Tibpromma et al. 2021).

##### ﻿ Nemania primolutea

Taxon classificationFungiXylarialesHypoxylaceae

Y.M. Ju, H.M. Hsieh and J.D. Rogers, Mycologia 97: 567 (2005)

645E4F85-C127-5F8C-B674-3EC6F4934DCF

Index Fungorum: IF501422

Facesoffungi Number: FoF17634

Fig. 8

###### Description.

*Endophytic* from healthy panicle of *Oryzasativa*. **Sexual morph**: Not observed. **Asexual morph**: Hyphomycetous. ***Conidiophores*** 1.5–4 µm wide, unbranched or sometimes dichotomously branched, smooth-walled, pale brown to hyaline. ***Conidiogenous cells*** 1.5–2.5 µm wide, cylindrical, geniculate, proliferating unclearly, smooth-walled, hyaline. ***Conidia*** 2.4–4.5 × 5.5–8.4 µm (*x̄* = 3.5 × 6.4 µm, *n* = 30), ellipsoid to obovoid, smooth, hyaline.

**Figure 8.**
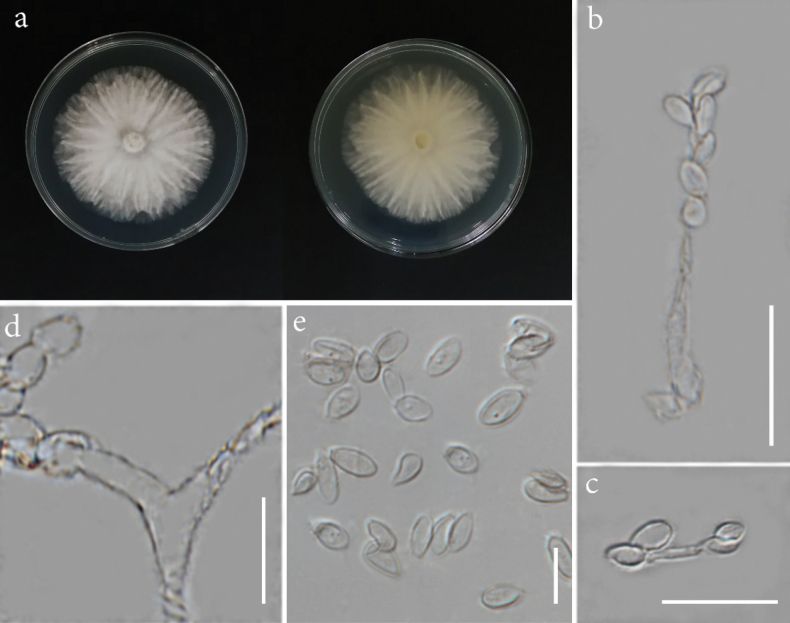
*Nemaniaprimolutea* (MFLUCC 24-0507, new host record). **a.** Front and reverse of the colony on PDA; **b, c.** Conidiophores and conidia; **d.** Branched conidiophores; **e.** Conidia. Scale bars: 20 μm (**b, c**); 10 μm (**d, e**).

###### Culture characteristics.

Colonies on PDA reaching 69–73 mm in diameter after 7 days at 28 °C, white, circular, velvety, slightly raised, with crenate margins; reverse yellowish white.

###### Material examined.

Thailand • Chiang Rai Province, Thoeng District, San Sai Ngam Sub-District, from healthy tissue part of rice panicle, 9 November 2021, Nootjarin Jungkhun (NS39-1a = MFLU 25-0026) (living culture MFLUCC 24-0507).

###### GenBank numbers.

MFLUCC 24-0507: ITS = PV241498, LSU = PV235235, *rpb*2 = PV275681, *tub*2 = PV275685.

###### Notes.

**Figure 9.**
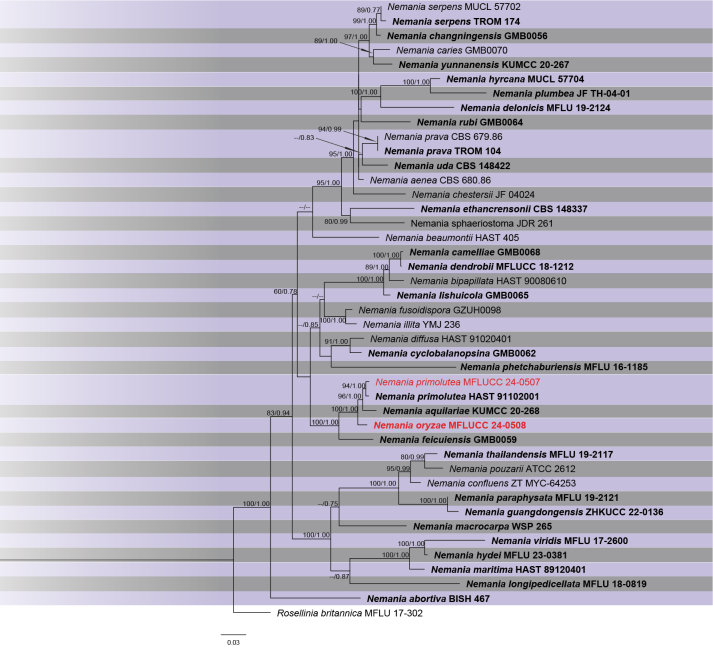
Phylogram of ML analysis based on combined ITS, LSU, *rpb*2, and *tub*2 sequence data. ML bootstrap support values equal to or higher than 60% and Bayesian probability values (PP) equal to or above 0.80 are given at the nodes (ML/PP). The tree is rooted to *Roselliniabritannica* (MFLU 17-302). The isolate from the current study is highlighted in red, and type strains are indicated in bold black.

The holotype strain of *Nemaniaprimolutea* (HAST 91102001) was isolated from a dead trunk of *Artocarpuscommunis* (Ju et al. 2005). According to the phylogram (Fig. 9), our strain was identified as *Nemaniaprimolutea* with 94% ML and 1.00 PP support. The isolate MFLUCC 24-0507 shares a similar asexual morph with HAST 91102001. However, our strain differs from HAST 91102001 in having larger conidia (2.4–4.5 µm × 5.5–8.4 µm in MFLUCC 24-0507 vs. 2.5–3 × 4.5–6.5 in HAST 91102001). This species has also been reported from *Lagerstroemia* sp. in Louisiana, USA (Garcia-Aroca et al. 2021), and *Ramalinaperuviana* (lichen) in the Philippines (Galinato et al. 2021). However, a lack of micro-morphology and insufficient molecular data were provided to verify the species identification. In this study, we illustrated *Nemaniaprimolutea* on rice as a new host record.

##### ﻿ Nemania oryzae

Taxon classificationFungiXylarialesXylariaceae

S. Absalan, S. Lumyong and K.D. Hyde sp. nov.

00C0EF49-7A72-52C3-ACF2-674821EEF4C3

Index Fungorum: IF903560

Facesoffungi Number: FoF17635

Fig. 10

###### Holotype.

MFLU 25-0027

**Figure 10.**
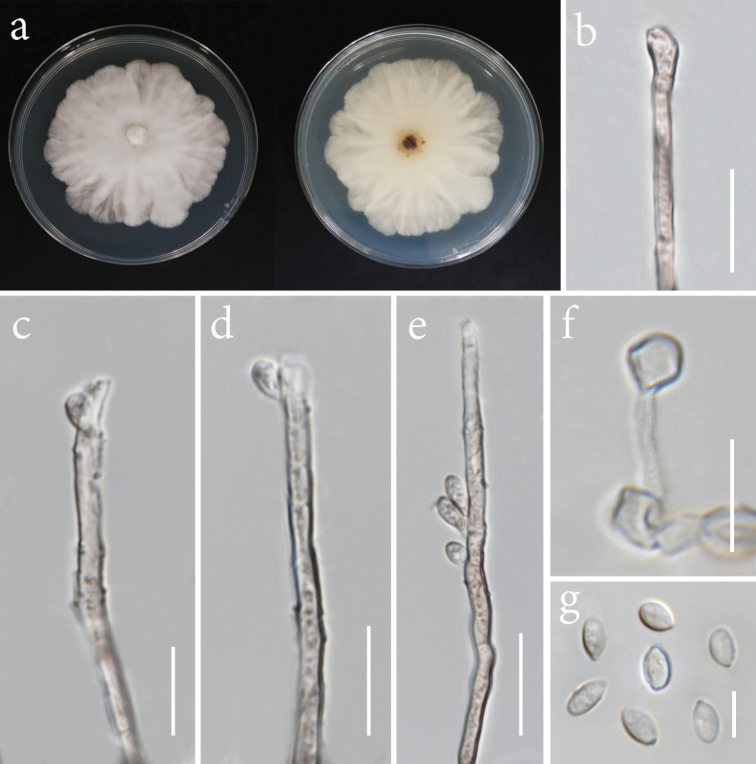
*Nemaniaoryzae* (MFLUCC 24-0508, ex-type). **a.** Front and reverse of the colony on PDA; **b–e.** Conidiophore and conidiogenous cells; **f.** Chlamydospore; **g.** Conidia. Scale bars: 10 μm (**b–g**).

###### Etymology.

*Oryzae* refers to the host genus *Oryza* from which it was isolated.

###### Description.

*Endophytic* from healthy panicle of *Oryzasativa*. **Sexual morph**: Not observed. **Asexual morph**: Hyphomycetous. ***Hyphae*** 1.5–2.5 µm wide, straight, branched, septate, hyaline. ***Conidiophores*** 3–3.5 µm wide, unbranched, septate, occasionally enlarged towards the upper part, pale brown to hyaline. ***Conidiogenous cells*** 2.5–3.5 µm wide, cylindrical, pale brown to hyaline. ***Conidia*** 4.5–7 × 3–4 µm (*x̄* = 6 × 3.5 µm, *n* = 20), obovoid, smooth, hyaline. ***Chlamydospores*** scarce, thick-walled, hyaline, globose to ellipsoidal.

###### Culture characteristics.

Colonies on PDA reaching 65–68 mm in diameter after 7 days at 28 °C, cotton white, circular, medium dense, velvety to felty, with undulate margins; reverse white.

###### Material examined.

Thailand • Chiang Rai Province, Phan District, from healthy tissue part of the rice panicle, 9 November 2021, Nootjarin Jungkhun (NS24-1a = MFLU 25-0027) (ex-type, living culture MFLUCC 24-0508).

###### GenBank numbers.

MFLUCC 24-0508: ITS = PV241499, LSU = N/A, *rpb*2 = PV275682, *tub*2 = PV275686.

###### Notes.

*Nemaniaoryzae* is proposed here as a new species based on multi-gene phylogenetic analyses. Our strain (MFLUCC 24-0508) clustered in a separate lineage, distinct from *N.primolutea*, *N.aquilariae*, and *N.feicuiensis*, with 100% ML and 1.00 PP bootstrap support (Fig. 9). A comparison of the nucleotide differences between *N.oryzae* and the closely related species, *N.aquilariae*, revealed 0.28% (across 357 nucleotides), 4% (across 886 nucleotides), and 5.2% (across 327 nucleotides) base pair differences in ITS, *rpb*2, and *tub*2 gene regions, respectively. *Nemaniaaquilariae* (KUMCC 20-0268) was isolated from the wood of *Aquilariasinensis* as an endophytic fungus and did not sporulate on the culture media (Tibpromma et al. 2021). Hence, morphological comparisons are not feasible due to the absence of micro-morphological details in *N.aquilariae’s* description. Sequence comparison between *N.oryzae* and *N.feicuiensis* showed base pair differences of 2.5% (across 357 nucleotides), 9.2% (across 886 nucleotides), and 13.4% (across 327 nucleotides) in the ITS, *rpb*2, and *tub*2 gene regions, respectively. Morphological distinction between the two species is also not feasible, as *N.feicuiensis* has only been documented as a sexual morph (Pi et al. 2021).

## ﻿Discussion

Rice is a vital food source, providing both protein and energy to more than half of the global population (Khush 2005). As an economically important crop, the majority of research has focused on its pathogens in efforts to improve yield (Ou 1985; Mew et al. 2004; Zheng et al. 2013; Saleh et al. 2014; Gupta et al. 2015; Bregaglio et al. 2016; Kongcharoen et al. 2020). As a result, the diversity of endophytic fungi in rice remains underexplored (Yuan et al. 2007; Naik et al. 2009; Rodriguez et al. 2009). Nevertheless, some studies have investigated endophytic fungi inhabiting healthy rice tissues in Thailand and other parts of the world (Wang et al. 2015, 2016; Seephueak et al. 2019; Ramaiah et al. 2020; Tian et al. 2021b). Previous reports have shown that members of Xylariomycetidae are among the least commonly encountered endophytes in rice, whereas *Penicillium*, *Aspergillus*, and *Fusarium* are among the most frequently isolated genera. Following these, *Curvularia*, *Trichoderma*, and *Cladosporium* are also frequently identified (Petrini 1991; Tian et al. 2004; Zakaria et al. 2010; Suada et al. 2012; Wang et al. 2015; Leewijit et al. 2016; Wijesooriya and Deshappriya 2016; Potshangbam et al. 2017; Seephueak et al. 2019; Roy et al. 2021; Li et al. 2022).

In the present study, eight endophytic xylariaceous strains were isolated from rice plants in northern Thailand. Identification was based on phylogenetic analyses in combination with morphological observation. However, morphological identification of endophytic xylariaceous fungi is often difficult due to their sterility or the limited diagnostic features of asexual morphs produced on standard media (Stadler et al. 2013). Among the six species identified, *Daldiniaeschscholtzii* has been previously reported from rice in Thailand and other Asian countries (Su-Han et al. 2019; Syamsia et al. 2021; Roy et al. 2023; Nishi et al. 2024). Su-Han et al. (2019) isolated *D.eschscholtzii* from the roots and leaves of two rice cultivars (RD47 and PT1) in central Thailand. In the present study, this species was isolated from panicles of three different rice cultivars from northern Thailand (Table 2). These findings suggest that *D.eschscholtzii* may be one of the most common endophytic species associated with rice, particularly in Thailand. Given that *D.eschscholtzii* is known to produce a wide array of secondary metabolites and exhibits antagonistic activity against plant pathogens, its prevalence in rice warrants further investigation (Wang et al. 2015; Shylaja et al. 2018; Yang et al. 2018; Liao et al. 2019; Lin et al. 2019; Zhang et al. 2019; Sibero et al. 2020; Khruengsai et al. 2021; Chen et al. 2024).

Endophytic species of *Nemania* have been reported from various hosts, including *Asparagopsistaxiformis* (red alga), *Torreyataxifolia* (Florida torreya), *Aquilariasinensis* (incense tree), *Taxusbaccata* (Iranian yew), and *Vitis* spp. (grapevine), and are recognized for their production of bioactive compounds (Ibrahim et al. 2017; Farsi and Farokhi 2018; Kumarihamy et al. 2019; Medina et al. 2019; Tibpromma et al. 2021). To the best of our knowledge, an association between *Nemania* and rice has not been previously reported (Farr and Rossman 2025). In this study, we identified one new species and one new host record of *Nemania* from two distinct rice cultivars. Unlike *Nemania*, species of *Microdochium* have frequently been reported on rice from countries such as Argentina, Ivory Coast, Japan, Sri Lanka, and the United Kingdom (Samuels and Hallett 1983; Gutiérrez et al. 2008; Hernández Restrepo et al. 2016; Pathmanathan et al. 2022). While we identified a new species of *Microdochium* from a healthy leaf in this study, species in this genus are also known to cause disease on grasses and cereals (Wen et al. 2015; Liang et al. 2019; Abdelhalim et al. 2020; Gagkaeva et al. 2020). On the other hand, *Microdochium* species have demonstrated antagonistic activity and the ability to produce beneficial secondary metabolites as endophytes (Helaly et al. 2018; Shadmani et al. 2018; Gavrilova et al. 2020).

Species of *Apiospora* are cosmopolitan (Crous and Groenewald 2013; Jiang et al. 2018; Wang et al. 2018) and exhibit diverse ecological lifestyles (Zhao et al. 1990; Martínez-Cano et al. 1992; Chen et al. 2014; Dai et al. 2017; Tian et al. 2021a; Ma et al. 2022; Liu et al. 2023; Zhang et al. 2023), occurring on a wide variety of hosts, with highest prevalence reported in members of the Poaceae (Mavragani et al. 2007; Dai et al. 2017; Pintos and Alvarado 2021; Zeng et al. 2022; Zhao et al. 2023). However, *Apiospora* species have rarely been reported from rice, with only a single record of *Apiosporarasikravindrae* (Rana et al. 2017). Here, we describe and illustrate two new host records of *Apiospora* associated with rice.

Although xylariaceous endophytes have been recorded from various host plants in many countries, including Thailand, their association with rice has been relatively overlooked. Given the ecological and biotechnological importance of this fungal group, future studies should continue to explore xylariaceous diversity in rice to uncover novel species, their ecological roles, and potential applications in agriculture, biotechnology, and medicine. In particular, investigating the functions of beneficial endophytic fungi could contribute to sustainable agricultural practices, including soil health improvement and enhanced crop productivity, while minimizing environmental impact.

## Supplementary Material

XML Treatment for Apiospora intestini

XML Treatment for Apiospora mukdahanensis

XML Treatment for Daldinia eschscholtzii

XML Treatment for Microdochium oryzicola

XML Treatment for Nemania primolutea

XML Treatment for Nemania oryzae

## ﻿Additional information

### Use of AI

No use of AI was reported.

### Author ORCIDs

Sahar Absalan https://orcid.org/0000-0002-6052-5381

Alireza Armand https://orcid.org/0000-0003-2455-3796

Ruvishika S. Jayawardena https://orcid.org/0000-0001-7702-4885

Nakarin Suwannarach https://orcid.org/0000-0002-2653-1913

Saisamorn Lumyong https://orcid.org/0000-0002-6485-414X

Kevin D. Hyde https://orcid.org/0000-0002-2191-0762

### Data availability

All of the data that support the findings of this study are available in the main text.
